# Genomic Investigation into the Virulome, Pathogenicity, Stress Response Factors, Clonal Lineages, and Phylogenetic Relationship of *Escherichia coli* Strains Isolated from Meat Sources in Ghana

**DOI:** 10.3390/genes11121504

**Published:** 2020-12-14

**Authors:** Frederick Adzitey, Jonathan Asante, Hezekiel M. Kumalo, Rene B. Khan, Anou M. Somboro, Daniel G. Amoako

**Affiliations:** 1 Department of Veterinary Science, University for Development Studies, P. O. Box TL 1882, Tamale 00233, Ghana; 2 Antimicrobial Research Unit, Infection Genomics and Applied Bioinformatics Division, University of KwaZulu-Natal, Durban 4000, South Africa; josante33@gmail.com; 3 School of Laboratory Medicine and Medical Science, University of KwaZulu-Natal, Durban 4000, South Africa; kumaloh@ukzn.ac.za (H.M.K.); myburgr@ukzn.ac.za (R.B.K.); anou.somboro@gmail.com (A.M.S.)

**Keywords:** genomics, virulome, stress response, clonality, phylogenetic analysis, *E. coli*, meat

## Abstract

*Escherichia coli* are among the most common foodborne pathogens associated with infections reported from meat sources. This study investigated the virulome, pathogenicity, stress response factors, clonal lineages, and the phylogenomic relationship of *E. coli* isolated from different meat sources in Ghana using whole-genome sequencing. Isolates were screened from five meat sources (beef, chevon, guinea fowl, local chicken, and mutton) and five areas (Aboabo, Central market, Nyorni, Victory cinema, and Tishegu) based in the Tamale Metropolis, Ghana. Following microbial identification, the *E. coli* strains were subjected to whole-genome sequencing. Comparative visualisation analyses showed different DNA synteny of the strains. The isolates consisted of diverse sequence types (STs) with the most common being ST155 (*n* = 3/14). Based Upon Related Sequence Types (eBURST) analyses of the study sequence types identified four similar clones, five single-locus variants, and two satellite clones (more distantly) with global curated *E. coli* STs. All the isolates possessed at least one restriction-modification (R-M) and CRISPR defence system. Further analysis revealed conserved stress response mechanisms (detoxification, osmotic, oxidative, and periplasmic stress) in the strains. Estimation of pathogenicity predicted a higher average probability score (P_score_ ≈ 0.937), supporting their pathogenic potential to humans. Diverse virulence genes that were clonal-specific were identified. Phylogenomic tree analyses coupled with metadata insights depicted the high genetic diversity of the *E. coli* isolates with no correlation with their meat sources and areas. The findings of this bioinformatic analyses further our understanding of *E. coli* in meat sources and are broadly relevant to the design of contamination control strategies in meat retail settings in Ghana.

## 1. Introduction

*Escherichia coli* (*E. coli*) are Gram-negative, rod shape, facultative anaerobic bacteria of the Enterobacteriaceae family [1,2]. They are naturally found in the gastrointestinal tract of animals and their droppings [2,3], and can cross-contaminate meat, especially under faulty processing conditions [4]. *E. coli* are classified into two major groups, namely, intestinal or diarrheagenic *E. coli* (DEC) and extraintestinal *E. coli*. DEC is divided into six pathotypes, namely, Enteropathogenic *E. coli*, Enterohaemorrhagic *E. coli*, Shiga toxin producing *E. coli*, Enteroinvasive *E. coli*, Enteroaggregative *E. coli*, Enterotoxigenic *E. coli*, Verotoxigenic *E. coli*, and diffusely adherent *E. coli* [5]. The extraintestinal *E. coli* group includes subtypes, such as neonatal meningitis associated *E. coli*, uropathogenic *E. coli*, and sepsis-associated *E. coli* [6,7]. Their various virulence genes differentiate these pathotypes. Their pathogenesis is initiated by the adherence to the host’s epithelial cell, and, then colonization with some pathotypes invaded the cells, thereby establishing infections [8].

*E. coli* survive by overcoming stressors released by their host or substrate [9]. In meats, *E. coli* must overcome stressors such as heat, especially during cold storage under refrigerated conditions, starvation, and acid when meats are treated with ingredients/spices for preservation and cooking. Stressors result in the release of regulator proteins and genes by *E. coli* to confer resistance and to promote survival [10,11]. This resistance and tolerance can lead to enhanced virulence.

*E. coli* of meat origin harbouring virulence genes have been reported [12,13,14]. Virulence genes can increase the pathogenicity of *E. coli* by means such as inhibition/evasion of the immune system of the host and obtaining its nutrition from the host, thereby, depriving the host of some nutrients [15]. Caruso et al. [14] found that 28% of extraintestinal *E. coli* of meat origin harboured virulence genes, which included *kpsMII, iutA*, and *PapA* genes. Lyhs et al. [13] characterised 207 *E. coli* isolates from poultry meat products and reported that a virulent gene was present in each isolate. In addition, all *E. coli* strains from raw meat and shell fish contained at least one virulence gene of the 16 genes detected [12].

Meat is any edible part of a slaughtered animal. It can be obtained from cattle, goat, sheep, poultry, and pigs, among other animals. Meat is an important source of protein and other nutrients for humans worldwide [16,17]. In Ghana, meat is an essential component of the diet of most people [18] and its association with pathogens represent a risk to public health. The most frequent mode of transmission for *E. coli* to man is through the consumption of food and water [19]. Meat has been implicated in several foodborne outbreak infections. Outbreaks of *E. coli* linked to ground beef and poultry have also been reported globally [20,21,22].

Molecular investigations have been carried using a number of typing techniques including enterobacterial repetitive intergenic consensus (ERIC), random amplified polymorphic deoxyribonucleic acid (RAPD), repetitive extragenic palindromic (REP), pulse-field gel electrophoresis, and multi-locus sequence typing [23,24,25,26,27]. Recent advances have promoted the use of whole-genome sequencing and metagenomics. Nonetheless, whole-genome sequencing (WGS) of pathogens has become the reference standard due to its accessibility and affordability, and has revolutionised the field of outbreak investigations [28]. WGS enables the genome of a bacterium to be sequenced and helps in the in silico prediction of various traditional typing methods, which explore short DNA strands with a quick turnaround time comparatively [29]. Reports on the genomic characterisation of *E. coli* from meat sources in Ghana are limited. The study used whole-genome sequencing to identify virulome, pathogenicity, stress response factors, clonal lineages, and phylogenomic relationship of *E. coli* isolated from various meat types in Ghana. This will provide useful information on the risk factors related to meat consumption and adaptive characteristics of *E. coli* that favour their clonal spread and survival of various niches.

## 2. Materials and Methods

### 2.1. Sample Collection and Identification of E. coli

#### 2.1.1. Sample Collection

A total of two hundred and twenty-five (225) meat samples comprising of beef (*n* = 45), chevon (*n* = 45), chicken (*n* = 45), guinea fowl (*n* = 45), and mutton (*n* = 45) were sampled in Tamale (Ghana) between April and December 2016. Sterile cotton swabs were used to swab an area of 10 cm^2^ of each meat sample. The swabs were transported under 4 °C and analysed immediately upon reaching the laboratory.

#### 2.1.2. Isolation of *E. coli*

The procedure in the Food and Drug Administration-Bacteriological Analytical Manual [2] with slight modification was used. The swabs were dipped into 10 mL of Buffered Peptone Water and incubated at 37 °C for 24 h. Afterward, 0.1 mL of the aliquots were streaked on Levine’s Eosin-methylene Blue Agar and incubated at 37 °C for 24 h. Presumptive *E. coli* colonies appeared as dark-centered and flat with or without metallic sheen. Presumptive *E. coli* colonies were purified on Trypticase Soy Agar and incubated at 37 °C for 24 h. They were identified and confirmed using Gram staining, growth on MacConkey Agar, growth in Brilliant Green Bile Broth, and the *E. coli* latex agglutination test. All media and reagents used were purchased from Oxoid Limited, Basingstoke, UK. A total of 189 *E. coli* isolates were recovered from the various meat sources (Table S1).

### 2.2. Selection of Isolates for Genome Sequencing and Assembly

A combination of cluster and simple random sampling were used to ensure that 14 isolates from the different meat sources and areas were chosen for WGS. A Genomic DNA (gDNA) of the *E. coli* isolates was extracted and purified using the QIAamp^R^ DNA Mini Kit (QIAGEN, Hilden, Germany). Following extraction, DNA quantification and the quality check was performed on a Qubit^®^ 2.0 fluorometer (Life Technologies, Carlsbad, CA, USA) and Nanodrop 8000 (Thermo Scientific, Waltham, MA, USA), respectively [30]. The Nextera XT DNA Sample Preparation Kit was used to prepare the paired-end library and WGS was performed using the Illumina MiSeq machine (Illumina, San Diego, CA, USA). The obtained raw reads were de-novo assembled with the Shovill pipeline version 0.9.0 that uses SPAdes version 3.11.0. The assembled contigs were deposited in GenBank under project number PRJNA484345.

### 2.3. Genome Annotation and Visualisation

The Pathogen watch platform was used to confirm the identity of the *E. coli* isolates generated from the WGS data [31]. The genomes of the strains were visualised using the GView Server [32]. The National Center for Biotechnology Information (NCBI) Prokaryotic Genome Annotation Pipeline (PGAP; version 4.3) [33] and Rapid Annotation using Subsystem Technology (RAST) Server (version 2.0) [34] were used for annotation of the isolates.

### 2.4. WGS-Based Molecular Typing of Isolates

Multilocus sequence typing (MLST) v2.0 (https://cge.cbs.dtu.dk/services/MLST/) was used to predict the sequence types (STs) from the assembled genomes. An eBURST [35] analysis was done in the MLST database (https://pubmlst.org/escherichia/) to find out whether the obtained STs were satellite clones, double-locus variant (DLV), or single-locus variant (SLV) of the known STs using the Achtman scheme. The reference *E. coli* online platform, CHTyper v1.0 (https://cge.cbs.dtu.dk/services/CHTyper/) was used to infer the FumC and FimH types.

### 2.5. Detection of the Stress Response Mechanisms, CRISPR Array, and Restriction-Modification System (R-M System)

The CRISPRCasFinder available at: https://crisprcas.i2 bc.paris-saclay.fr/CrisprCasFinder/Index [36] was used to infer common CRISPR and Cas loci in the assembled genomes. The Restriction Modification Finder at: https://cge.cbs.dtu.dk/services/Restriction-ModificationFinder/ and Pathosystems Resource Integration Center (PATRIC) platform (https://www.patricbrc.org/) were used to predict the restriction modification system in the isolates.

### 2.6. Assessment of Pathogenic Potential

The automated mode of the PathogenFinder web service was used to estimate the pathogenic potential in the *E. coli* genomes [37]. All *E. coli* isolates were subjected to the pathogenicity prediction using fasta formatted genome data.

### 2.7. Virulome Analysis

Virulence factors corresponding to main bacterial virulence determinants (toxin, adherence, motility, iron uptake, and secretion system) associated with *E. coli* were searched with the Virulence Finder 2.0 at: https://cge.cbs.dtu.dk/services/VirulenceFinder/ [38].

### 2.8. Comparative Phylogenomic Analysis and Metadata Insights

The CSI Phylogeny-1.4 (https://cge.cbs.dtu.dk/services/CSIPhylogeny-1.2) was used to infer the phylogenetic relationship between *E. coli* genomes [39]. The genome of *K. quasi-pneumoniae* strain P27-02 (accession number: NXHG00000000.1) served as the outgroup to root the tree for easy configuration of the phylogenetic distance between the isolates. The pipeline was run with default parameters. The phylogeny was viewed with annotations for metadata (virulome, sequence types, meat source, and area) using Phandango [40] to provide vital insights of the phylogenetic tree.

## 3. Results

### 3.1. E. coli Strains and Data Source for Comparative Genome Analysis

A total of 14 isolates obtained from five meat sources [beef (*n* = 3), chevon (*n* = 3), guinea fowl (*n* = 3), local chicken (*n* = 3), and mutton (*n* = 2)] and five areas [Aboabo (*n* = 2), Central market (*n* = 3), Nyorni (*n* = 3), Victory cinema (*n* = 2), and Tishegu (*n* = 4)] based in the Tamale Metropolis, Ghana (Table 1) were selected for further bioinformatic and comparative analyses. 

### 3.2. Genomic Visualisation and Annotation

**Figure 1:**
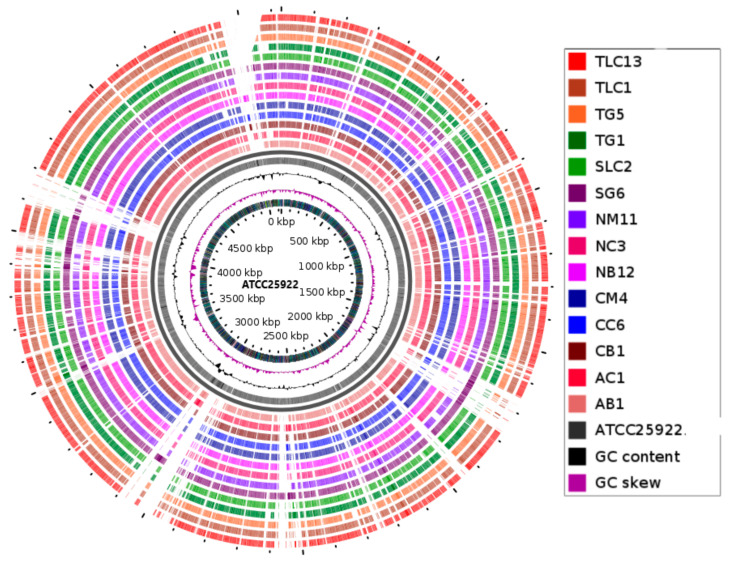
Comparative visualisation of the isolates (*n* = 14) with the *E. coli* reference strain (ATCC25922, Accession number: CP009074). The map was constructed using the GView online server (https://server.gview.ca/). The concentric circles represent comparisons between ATCC25922 and, starting with the inner circle, genome assemblies from *E. coli* genomes (strain ID: AB1, AC1, CB1, CC6, CM4, NB12, NC3, NM11, SG6, SLC2, TG1, TG5, TLC1, and TLC13). Colour codes are given for each strain with a synteny identity, ranging from 90–97%.

The global Pathogen watch platform confirmed the phenotypic identity of the *E. coli* genomes. Comparative visualisation analysis via the GView server showed different DNA synteny of the strains with a coverage range of 90–97% from the reference strain (ATCC25922) in all the *E. coli* genomes (Figure 1).

### 3.3. In Silico Typing of the E. coli Isolates

**Figure 2:**
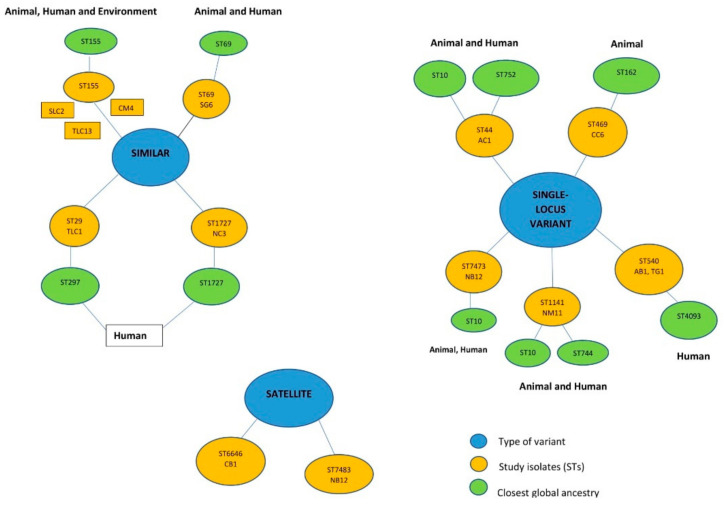
A graphical depiction showing the eBURST (Based Upon Related Sequence Types) analyses of the study sequence types with global curated STs in *Escherichia* PubMLST database using the Achtman scheme. eBURST identified four similar clones (have been reported), five single-locus variants (SLV), and two satellite lineages (more distantly) with global curated *E. coli* STs from animal (food), human, and environmental sources. The colour codes depicted the type of variant (blue), study isolates (STs) (orange), and closet global ancestry (green).

Eleven different sequence types were identified in the isolates (Table 1). The predominant sequence types were the ST155 (*n* = 3) and ST540 (*n* = 2). Of note, most of the sequence types were singletons (*n* = 9) (Table 1). There was high diversity in the clonal lineages of strains with respect to their source and area of origin (Table 1). Based Upon Related Sequence Types (eBURST) analyses of the study sequence types identified four similar clones, five single-locus variants (SLV), and two satellite clones (more distantly) with global curated *E. coli* STs from animals (food), humans, and environmental sources (Figure 2 and Table S3). The *E. coli* strains possessed diverse serotypes with no association with the meat sources, area, and sequence types (Table 1). Of note, not all the O-antigen and H-antigen loci in the strains were predicted. The FumC and FimH types were identified in the *E. coli* isolates, which correlated with the sequence types to a large extent except for two isolates (TG5 and CM4) that differed in their STs and FumC/FimH types (Table 1). The FumC7/FimH54 (*n* = 3) and FumC4/FimH32 (*n* = 2) were most frequently identified. Several singletons FumC and FimH types were also identified.

### 3.4. Genomic Prediction of Defence Systems (CRISPR-Cas Elements and R-M System) and Stress Response Factors

**Figure 3:**
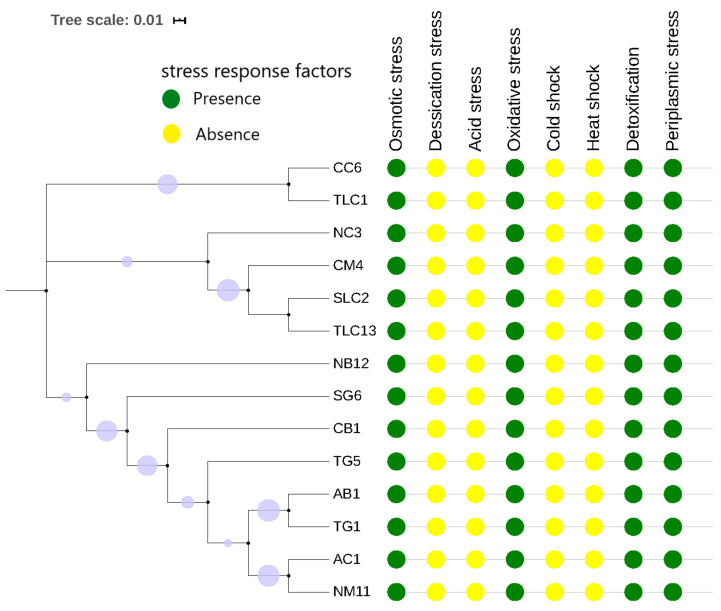
A colour gradient generated phylogenetic tree of distribution of stress response factors across the *E. coli* isolates (*n* = 14). The green colour represents the presence of the gene, and the yellow colour represents the absence of the gene. Conserved stress response factors (osmotic stress, oxidative stress, detoxification, and periplasmic stress) were found in all the strains irrespective of their sources, area, and sequence types.

The genomes of the strains contained CRISPR arrays (*n*_range_ = 3–9) with a Cas element belonging to the same subcategory (Type1 E) except for two isolates, known as CB1 (Beef from Central Market) and TG5 (Guinea fowl from Tishegu) that did not encode any Cas element (Table 1). All the isolates possessed at least one restriction modification (R-M) system within the genome. The Type II R-M system was the most common in the *E. coli* isolates with *M.EcoKII* being the most dominant (*n* = 12), which is followed by *M.EcoJA03 PDcm* (*n* = 7). The isolate SG6 obtained from Guinea fowl (Victory Cinema) contained the highest number of R-M systems (*n* = 3, Type I, II, and IV) (Table 1). Conserved stress response factors including the detoxification system, osmotic, oxidative, and periplasmic stress were found in all the isolates (Figure 3). The oxidative stress response was the most predominant mechanism (*n* = 7) and putative stress response genes/proteins (*n* = 38) in the isolates.

### 3.5. Pathogenic Potential and Putative Virulence Factor Predictions

**Figure 4:**
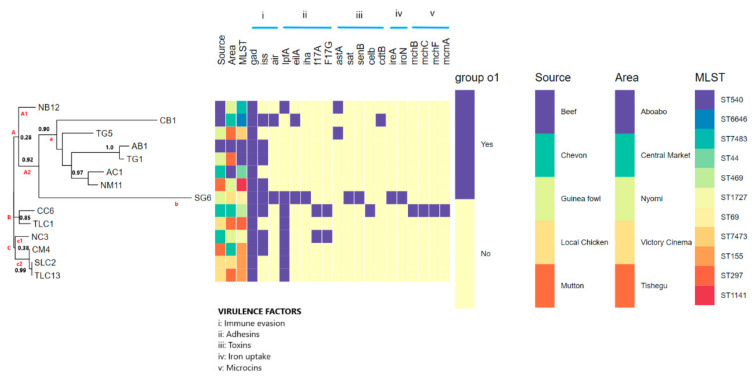
A phylogenomic branch coupled with metadata of isolate information (source, area, sequence types (STs), and virulence factors) showing the relationship between the study isolates (*n* = 14) using Phandango. The colour codes differentiated the different sources, areas, and STs. The virulence factors are represented by Roman numerals I: immune evasion, II: adhesins, III: toxins, IV: iron uptake, V: microcins.

The genomes of the *E. coli* predicted a 0.937 mean probability (P_score_) of being a human host-pathogen and was found to match pathogenic families ranging between 525 and 1001 (Table 1). The whole-virulome analysis predicted differences in the virulence factors possessed by the isolates. Nineteen putative virulence genes of the five main classes (adhesins, toxins, immune evasion, iron uptake, and microcin) of *E. coli* (Table 1 and Figure 4). Of note, adhesins (*n* = 5 genes) and toxins (*n* = 5 genes) were the most predominant virulence factor class. The most common virulence factors were *gad* (*n* = 14), *iss* (*n* = 8), and *lpfA* (*n* = 8), with *eilA*, *iha, cdtB*, *mchB, mchC, mchF*, and *mcmA* occurring in single isolates (Table 1 and Figure 4). The isolates CC6 and SG6 harbored the highest number of genes while all the isolates contained at least one virulence gene. As shown in Figure 4, diverse virulence genes that were mostly clonal-specific were identified. A specimen source-virulome association comparison was made (Figure 4) and there was little evidence to suggest that strains from a specific source had more virulence genes than others.

### 3.6. Phylogenomic Analysis

The tree analyses via the whole genome sequencing single nucleotide polymorphism (WGS SNP) tree differentiated the strains into three clusters (A, B, and C) with each cluster containing sub-clusters (Figure 4). Cluster A had the largest number of isolates (*n* = 9). The *E. coli* isolates showed significant phylogenetic diversity (Figure 4) with the phylogenomics showing greater resolution than the MLST technique. For instance, the sub-cluster C2 contained isolates (CM4, SLC2, and TLC13) from the same clone (ST155), but phylogenetically found on different sub-branches. The tree analyses coupled with metadata showed no patterns among the isolates with respect to their sources and areas (Figure 4).

## 4. Discussion

*E. coli* are among the most common foodborne pathogens associated with infections reported from meat sources. However, information on the virulome, pathogenic potential, and adaptive characteristics associated with its persistence using high throughput technologies in Africa are lacking. Therefore, this study investigated the virulome, pathogenicity, stress response factors, clonal lineages, and the phylogenomic relationship of 14 *E. coli* isolated from different meat types in Ghana using WGS. Of the 14 isolates, eight (57.1%) were multi-drug resistance (defined as resistance to three or more different classes of antibiotics) and possessed multiple antibiotic resistome [41].

The GView Server was used for the comparative circular visualisation of the multiple genomes to explore the inter-relationship between the genomic data points [32]. The circular plots generated for genomic data is tailored to gain the best biological insights into the data [42]. The inspection of the generated circular maps revealed variations in the genomic synteny (90–97%) as well as distinct trends of majorly gapped regions from the reference (Figure 1), indicating a possible closer and diverse association between the study strains.

The diversity of sequence types identified in the *E. coli* isolates is consistent with findings reported from China, USA, Korea, and Ghana [43,44,45,46]. Whiles four sequence types, namely ST155, ST69, ST29, and ST1727, found in the study have already been reported in human, animal, and environmental samples globally [43,47,48]. Two sequence types known as ST6646(CB1) and ST7483(NB12) were not associated with any globally curated ancestry lineages (satellite-variant) (Figure 2). Interestingly, globally reported ST10 (*n* = 3/5) was the most common ancestry (single-locus variant) for the five remaining sequence types (ST44, ST469, ST540, ST1141, and ST7473) (Figure 2). The results of the FumC-FimH types corroborated with the MLST scheme and affirmed by the high genetic diversity between the isolates could be reliable to indicate the degree of clonality between *E. coli* strains.

The CRISPRCas defence system that controls the adaptive immunity mechanisms responsible for protecting bacteria against viral invasion was predicted in silico (Table 1) [49,50]. The CRISPRCas has been advanced as a critical tool for the survival of bacteria in the microbial environment. It is, thus, likely this system contributed to the survival of these strains in different settings [49,51]. Additionally, the Type I-E CRISPR/Cas cluster detected in the strains has been reported to be involved in transcription regulation of endogenous genes with a possibility to rewire the regulatory interactions with the selection of targets done through native adaptation [52,53]. Further analysis showed other defence systems such as the restriction-modification (R-M) types I, II, and IV offering protection against exogenous DNA and, thus, increasing their survival [54]. The detection of the 2 R-M systems in most strains was not unusual considering that about 12.5% of bacteria commonly harbour more than one R-M system [54]. Moreover, the presence of the Type II system (Table 1) in all the isolates affirmed its abundance in *E. coli* [54,55]. However, there is little information about its regulation and further studies should be done to understand its regulation.

Bacteria deal with continuous stress and, thus, develop mechanisms to tolerate different stressors and adapt to hostile micro-environments [9]. Several conserved cellular mechanisms aiding in the survival of these strains in extreme biological niches were identified (Figure 3 and Table S3). The *E. coli* isolates showed genomic features to control the pH level and osmolarity to adapt in the different environment. For instance, to withstand drastic exchanges in osmolarity, they contained molecules such as Aquaporin Z and glycerol uptake protein facilitator (*glpF*) found to play a vital role in water and glycerol movement, respectively [56,57] (Table S3). They also harboured the biosynthesis system (Osmoprotectant) and choline-glycine betaine uptake, which manage the feedback mechanism by regulating these molecules to control high salinity in their micro-environment. Moreover, they serve as sources of nitrogen and carbon for the isolates to grow [58]. In the case of oxidative stress, the strains contained enzymes (peroxidase enzyme, reductase, and super-oxidase dismutase) that can neutralise harmful products such as the reactive oxygen species (ROS) before damaging vital cellular components, such as proteins, DNA, and membrane lipids (Figure 3 and Table S3) [59]. The periplasmic tolerant response mechanisms reported to aid Enterobacteria in surviving acidic environments were found in the strains (Table S3) [60,61]. More so, the periplasmic membrane proteases (*RasP/YluC*) involved in the splitting of anti-sigma factor regulons (*RpoE*/*RseA*) to prevent protein misfolding caused in cell stress factors were found in the isolates [62]. The *E. coli* strains were armed against toxicants such as the harboured detoxification proteins (*YbaT*, *CysA*, *DedA*, and *TehA/TehB*) to fight the harmful effects on proteins caused by heavy metals (tellurite and selenite) in the microenvironment (Figure 3 and Table S3) [63,64]. However, biology bacterial stress response mechanisms are still part of on-going research. Therefore, more studies will be needed to ascertain the exact mechanisms controlling the phenotypes of the stress response to aid in the discovery of potential targets for development of new anti-bacterial treatments.

The pathogenic potential (P_score_) with the probability ranging from 0 to 1 is employed in the automatic estimation of the chance of an isolate being a human pathogen [37,65]. This theoretical prediction of the pathogenicity using machine-learning algorithms to distinguish among commensal or pathogenic strains estimated a higher mean probability (P_score_ ≈ 0.937), implying the strains from non-human origin to be pathogenic to humans (Table 1). Nevertheless, given the complexity between host-pathogen interactions, other experiments (including cell culture assays) are required to find out the practical relation of this theoretical measure (pathogenic potential) as this machine learning algorithm can be overestimated and it should be interpreted with caution [66,67].

The diversity of the virulome shown in the isolates is concerning as they are involved in virulence and play a significant role in their survival and pathogenesis. The virulome patterns were clonal-specific. For instance, the iron acquisition genes (*ireA*, *iroN*) and microcin H47 system (*mcmA*, *mchB*, *mchC* and *mchF* genes) were only possessed by the ST469 (Figure 4). The iron-acquisition system enhances the successful persistence and pathogenesis of the strain in the host [68,69]. The presence of these iron-uptake genes is very worrying, as these genes have been reported in various pathogenic *E. coli* [70,71]. The microcin H47 system acts as a bactericidal antibiotic secreted under conditions of nutrient depletion and exerts potent antibacterial action against closely related species, offering a survival advantage for the ST469 strain [72,73]. The adhesin-encoding (*f17 A, f17 G*) genes known for causing intestinal and extraintestinal disease in animals and humans were found in ST469 and ST1727 [74]. More so, a 2014 study by Morgan et al. [75] reported the production of F17 fimbriae by pathogenic *E. coli* implicated in septicaemia and diarrhoea outbreaks in lambs and calves. Moreover, the clonal-specific putative toxins (*senB*, *sat*, *cdtB*, and *celb*) were also observed in the isolates (Figure 4). The toxins offer the strains the ability to mediate cell membrane damage, inducing the formation of cytokines, and killing or reducing neutrophils [76]. These toxins have been identified in some strains (Table 1 and Figure 4), and are associated with causing illness in animals and humans [77,78]. The similarity of the virulence determinants between clones from the diverse settings imply that they were mainly acquired vertically and not via horizontal gene transfer. Moreover, no association could be established between the specimen sources, areas, and the virulome.

The phylogenetic relationships of *E. coli* strains were analysed by WGS SNPs tree. The greater resolution power of WGS sequencing over MLST was shown by the clustering of similar STs on the different branches and in sub-clusters (Figure 4, sub-cluster C2), giving credence to the need to shift to genomic investigation for a better understanding of the evolution of pathogens. Furthermore, tree analyses coupled with metadata insights depicted the diversity among the *E. coli* isolates with no correlation with their meat sources and sampling areas. The use of visual analytics increases confidence during molecular investigation of pathogens [65,79,80].

The WGS data could not infer the different *E. coli* pathotypes owing to a multiplicity of factors, such as genes, under review following concerns about specificity and unknown essential virulence determinants for some pathotypes, which has been reported in literature [81,82]. Hence, not all isolates comply with the standard pathotyping scheme. Additionally, the upsurge of hybrid strains of *E. coli* pathotypes owing to the mobility of most of the genes that encode virulence makes it easy for gene sharing and causing problems of specificity [81,82]. Finally, the particular limitation of WGS pathotyping is its limited capacity to accommodate new strains that do not comply with known categories since the current nomenclature is unwieldy and inflexible. The current challenges should be resolved to harness the full capacity of WGS to aid our understanding of the pathobiology and evolution of this highly versatile and adaptable *E. coli* species from different settings.

## 5. Conclusions

The findings of this present study revealed the diverse clonal lineages in the *E. coli* isolates with a rich repertoire of defence systems, conserved battery of stress tolerant factors, highly predicted pathogenic potential, and clonal-specific putative virulence factors in various meat types within the Tamale metropolis of Ghana, which is worrying for meat contamination management. Phylogenomic analyses combined with a metadata confirmed this high genetic diversity with no correlation with their meat sources and areas. Further molecular surveillance studies involving a large data set are recommended to help food safety and public health practitioners in the design of contamination control strategies in meat retail settings in Ghana.

## Figures and Tables

**Table 1:** Summary of the population, the source of specimens, sample type, and genotypic characteristics of the isolates.

1 D	Demographic Information		In Silico Typing				Molecular Defence Systems		Pathogenic Determinants	
	Source	Area	MLST	Serotype ^1^	CHTyper		CRISPRs (Cas) (Cluster)	Restriction-Modification (R-M) System	Pathogenicity Score (Pathogenic Families)	Virulence Factors
					FumC	FimH				
AB1	Beef	Aboabo	540	O11	fumC7	fimH54	7 (1) CAS-TypeIE	Type II (*M.EcoGVI, M.Eco4255 Dcm*) Type IV (*EcoAPECGmrSD)*	0.923 (525)	*gad, iss*
CB1	Beef	Central Market	6646	H39	fumC203	fimH562	3 (0)	Type I (*S.Sso30807 I)* Type II (*M.EcoKII, M.EcoGI, M.Eco3609 Dcm)*	0.938 (698)	*air, cdtB, eilA, gad, iss*
NB12	Beef	Nyorni	7483	O8	fumC23	fimH38	3 (1) CAS-TypeIE	Type I (*S.EcoNIH1 II, EcoO127 I, M.EcoNIH1 II*) Type II (*M.EcoKII, M.Eco3740 Dcm)*	0.942 (710)	*astA, gad, lpfA*
AC1	Chevon	Aboabo	44	O162	fumC11	fimH54	8 (1) CAS-TypeIE	Type I (*M.Eco6409 I, S.Eco3325 I, M.Eco2747 II*) Type II (*M.EcoGVII, M.EcoKII, M.EcoJA03 PDcm)*	0.938 (698)	*gad*
CC6	Chevon	Central Market	469	O8	fumC65	fimH68	6 (1) CAS-TypeIE	Type I (*M.Sso30807 I),* Type II (*M.EcoKII, M.Eco3740 Dcm)*	0.940 (678)	*celb, f17 A, f17 G, gad, iss, lpfA mchB, mchC, mchF, mcmA*
NC3	Chevon	Nyorni	1727	O88	fumC19	fimH31	3 (1) CAS-TypeIE	Type II (*M.EcoKII, M.EcoGVII, M.EcoJA03 PDcm)*	0.940 (686)	*f17 A, f17 G, gad, iss, lpfA*
SG6	Guinea fowl	Victory Cinema	69	O15	fumC35	fimH27	6 (2) CAS-TypeIE	Type I (*EcoKI, M.EcoJA69 PI, S.EcoJA69 PI),* Type II (*M.EcoGI, M.EcoKII, M.Eco3317 Dcm)* Type IV *(EcoKMrr)*	0.930 (1001)	*air, eilA, gad, iha, ireA, iroN, iss, IpfA, sat, senB*
TG1	Guinea fowl	Tishegu	540	O9	fumC7	fimH54	8 (1) CAS-TypeIE	Type II (*M.EcoGVII, M.EcoJA03 PDcm)*	0.934 (564)	*gad, iss*
TG5	Guinea fowl	Tishegu	7473	O61	fumC7	fimH54	10 (0)	Type II (*M.EcoKII, M.EcoJA03 PDcm)*	0.929 (604)	*astA, gad*
SLC2	Local Chicken	Victory Cinema	155	H9	fumC4	fimH32	4 (1) CAS-TypeIE	Type II (*M.EcoKII, M.EcoGVII, M.EcoJA03 PDcm)*	0.943 (651)	*gad, lpfA*
TLC1	Local Chicken	Tishegu	297	H9	fumC65	fimH38	8 (1) CAS-TypeIE	Type I (*M.Eco3609 I)* Type II (*M.EcoKII, M.EcoGI, M.Eco3609 Dcm)*	0.943 (691)	*gad, lpfA*
TLC13	Local Chicken	Tishegu	155	O132	fumC4	fimH32	4 (1) CAS-TypeIE	Type II (*M.EcoKII, M.EcoGVII, M.EcoJA03 PDcm)*	0.943 (648)	*gad, lpfA*
CM4	Mutton	Central Market	155	H40	fumC4	fimH366	6 (1) CAS-TypeIE	Type I (*M.Eco2747 II, EcoR124 II),* Type II (*M.EcoE1140 Dcm, M.EcoKII)*	0.940 (655)	*gad, iss, lpfA*
NM11	Mutton	Nyorni	1141	O113	fumC11	fimH25	9 (1) CAS-TypeIE	Type I (*M.EcoJA65 PI*), Type II (*M.EcoJA03 PDcm*, *M.EcoKII*)	0.936 (623)	*gad, iss*

**^1^** Not all the O-antigen and H-antigen loci in the strains were predicted.

## Supplementary Materials

The following are available online at https://www.mdpi.com/2073-4425/11/12/1504/s1. Table S1. Prevalence of *E. coli* in meat samples sold at the Tamale Metropolis. Table S2. A table showing the eBURST (Based Upon Related Sequence Types) analyses of the study sequence types with global curated STs in *Escherichia* PubMLST database. Table S3. In silico identification and characterisation of conserved stress response mechanisms in the *E. coli* strains.
