# Supplementary material for: Genomic Investigation into the Virulome, Pathogenicity, Stress Response Factors, Clonal Lineages, and Phylogenetic Relationship of Escherichia coli Strains Isolated from Meat Sources in Ghana
Source: Genes (Basel). 2020 Dec 14;11(12):1504. doi: 10.3390/genes11121504 (PMC7764966; doi:10.3390/genes11121504)
Supplement: Supplementary file 1 [file genes-11-01504-s001.pdf]

Table S1. Prevalence of *E. coli* in meat samples sold at the Tamale Metropolis.

Sample	No. of samples examined	[a]No. samples positive	[b]No. *E. coli*
Beef	45	39	39
Chevon	45	34	34
Mutton	45	40	40
Local chicken	45	36	36
Guinea fowl	45	40	40
Overall	225	189	189

[a]Number of samples positive for *E. coli.*

[b]One *E. Coli* isolate was selected from each positive sample.

Table S2. A table showing the eBURST (Based Upon Related Sequence Types) analyses of the study sequence types with global curated STs in *Escherichia* PubMLST database.

MLST (Isolate)	Type of clone	Closet global ancestry sequence type (ST)	Source
ST69 (SG6)	Similar [a]	ST69	Animal (Food), Human
ST155 (SLC2, TLC13, CM4)	Similar	ST155	Animal (Food), Human, Environment
ST297 (TLC1)	Similar	ST297	Human
ST1727 (NC3)	Similar	ST1727	Human
ST44 (AC1)	Single-Locus Variant (SLV) [b]	ST10, ST752	Animal (Food), Human
ST469 (CC6)	Single-Locus Variant (SLV)	ST162	Food
ST540 (AB1, TG1)	Single-Locus Variant (SLV)	ST4093	Human
ST1141 (NM11)	Single-Locus Variant (SLV)	ST10, ST744	Animal (Food), Human
ST7473 (NB12)	Single-Locus Variant (SLV)	ST10	Animal (Food), Human
ST6646 (CB1)	Satellite [c]	None	-
ST7483 (NB12)	Satellite	None	-

[a] Similar: study isolate was similar to a global curated known sequence type.
[b] Single-Locus Variant (SLV): study isolate only shared similarity with global curated known sequence types that differed in one allelic gene.
[c] Satellite: study isolate as a distantly related and did not shared any similarity with global curated known sequence types.

Table S3. In silico identification and characterization of conserved stress response mechanisms in the *E. coli* strains.

Type of Mechanisms	Associated Proteins/Enzymes/Genes
**Osmotic stress (n=14)**	
a. Osmoregulation (4)	Outer membrane protein A precursor
	Aquaporin Z
	Osmotically inducible protein (*OsmY*)
	Glycerol uptake facilitator protein
b. Osmoprotectant ABC transporter (4)	Permease protein (*YehY*)
	Inner membrane protein (*YehW*)
	Binding protein (*YehZ*)
	ATP-binding subunit (*YehX*)
c. Synthesis of osmoregulated periplasmic glucans (3)	Glucans biosynthesis protein *C*
	Glucans biosynthesis protein *D* precursor
	Glucans biosynthesis protein *G* precursor
d. Choline and Betaine Uptake and Betaine Biosynthesis (3)	High-affinity choline uptake protein (*BetT*)
	Choline dehydrogenase
	Betaine aldehyde dehydrogenase
**Oxidative stress (n=38)**	
a. Protection from Reactive Oxygen Species (3)	Superoxide dismutase [Cu-Zn] precursor
	Cytochrome *c551* peroxidase
	Superoxide dismutase [Fe]
b. Oxidative stress (9)	Redox-sensitive transcriptional activator (*SoxR*)
	Superoxide dismutase [Cu-Zn] precursor
	Paraquat-inducible protein B
	Superoxide dismutase [Fe]
	Ferric uptake regulation protein FUR
	Superoxide dismutase [Mn]
	Nitrite-sensitive transcriptional repressor (*NsrR*)
	Paraquat-inducible protein A
	Fumarate and nitrate reduction regulatory protein
c. Glutathione: Biosynthesis and gamma-glutamyl cycle (3)	Gamma-glutamyltranspeptidase
	Glutathione synthetase
	Glutamate--cysteine ligase
d. Glutathione: Non-redox reactions (10)	Glutathione S-transferase
	Uncharacterized glutathione S-transferase-like protein
	Lactoylglutathione lyase
	FIG005121: SAM-dependent methyltransferase
	Glutathione S-transferase, omega
	Uncharacterized GST-like protein (*yncG*)
	Uncharacterized GST-like protein *yghU* associated with glutathionylspermidine synthetase/amidase
	Hydroxyacylglutathione hydrolase
	Probable glutathione S-transferase, *YfcF* homolog
	Probable glutathione S-transferase, *YfcG* homolog

	Glutathione S-transferase (EC 2.5.1.18)
e. Glutathione: Redox cycle (6)	Glutathione reductase
	Glutaredoxin-like protein (*NrdH)*, required for reduction of Ribonucleotide reductase class Ib
	Glutathione peroxidase
	Glutaredoxin 3 (*Grx3*)
	Glutaredoxin 1
	Glutaredoxin 2
f. Glutaredoxins (4)	Glutaredoxin-like protein (*NrdH*), required for reduction of Ribonucleotide reductase class Ib
	Glutaredoxin 3 (*Grx3*)
	Glutaredoxin 1
	Glutaredoxin 2
g. Glutathionylspermidine and Trypanothione (3)	Glutathionylspermidine amidohydrolase
	Uncharacterized GST-like protein *yghU* associated with glutathionylspermidine synthetase/amidase
	Glutathionylspermidine synthase
**Periplasmic stress (n=11)**	
a. Periplasmic Acid Stress Response in Enterobacteria (4)	Transcriptional activator (GadE)
	Membrane transporter (*HdeD*), H-NS repressed
	Chaperone (*HdeA*)
	Chaperone (*HdeB*)
b. Periplasmic Stress Response (7)	Sigma factor *RpoE* negative regulatory protein *RseA*
	Outer membrane stress sensor protease (*DegS*)
	Outer membrane protein H precursor
	Intramembrane protease (RasP/YluC)
	Sigma factor *RpoE* negative regulatory protein *RseB* precursor
	Outer membrane stress sensor protease *DegQ*, serine protease
	HtrA protease/chaperone protein
**Detoxification (n=9)**	
a. Uptake of selenate and selenite (3)	Inner membrane transport protein (*YbaT*)
	Sulfate and thiosulfate import ATP-binding protein (*CysA*)
	DedA protein
b. Tellurite resistance (Chromosomal determinants) (3)	Uncharacterized membrane lipoprotein clustered with tellurite resistance proteins (*TehA*/*TehB*)
	FIG005189: putative transferase clustered with tellurite resistance proteins (*TehA*/*TehB*)
	Tellurite resistance protein (*TehA*)
c. Glutathione-dependent pathway of formaldehyde detoxification (3)	S-formylglutathione hydrolase
	S-(hydroxymethyl)glutathione dehydrogenase
	FrmR: Negative transcriptional regulator of formaldehyde detoxification operon
